# Vesico-Vaginal Fistula—A New Operation Proposed, by Which the Twisted Suture May Be Applied

**Published:** 1860-02

**Authors:** Paul F. Eve


### VESICO-VIGINAL FISTULA,

### A NEW OPERATION PROPOSED, BY WHICH THE TWISTED SUTURE MAY BE APPLIED.

BY PAUL F. EVE, M. D.

I submit to the profession an instrument by which the operation for *vesico-viginal fistula*, may, I think, be greatly simplified. The difficulties I have always encountered in attempting to relieve this most distressing affection, and the want of success even by the recent American improvements, have led to its suggestion; and without waiting to try it in a case, not knowing when an opportunity will be offered, the method is at once presented for the consideration of others. In common with my professional brethren, I hailed with pride and pleasure the important practical additions made on this subject by our distinguished countrymen, Drs. Sims and Bozeman, and remarked at the time to a friend, when the *button suture* was announced,—it is perfection. Subsequent experience, however, has taught me that something more is still required to heal vesico-vaginal fistula, with much degree of facility or success, and that almost any proposition (promising amelioration in its treatment) would be favorably received.—And for proof that I am not singular in this respect, in the publications of those who have operated, nearly all have testified that it is a very painful, tedious, complicated and uncertain operation.

The plan proposed is by a *screw-clamp* so to project the edges of the fistula that it may be closed by the *twisted suture.*

This instrument consists of two thumb-screws six inches in length and a line in thickness, with the threads cut three inches one-half of the way right, and the other half left-handed, on each of them, so that two clamps through which they pass may be made to approach each other by turning the screws. It is made of polished steel, except the edges of the clamps which have small projecting teeth to prevent their gliding over the mucous surface of the vagina when applied to it. I have these put one-fourth of an inch apart, with a projection one-eighth of an inch slightly curved towards the fistula. The clamps themselves may be bent towards the vaginal surface.

The patient is placed upon her knees and elbows, with Dr. Sim's lever-speculum introduced, the screw clamp is then applied, with one of the clamps three-fourths of an inch anterior, and the other the same distance posterior to the fistula. While thus held in position, by means of a curved catheter or forceps passed through the urethra into the bladder, the fistulous opening is oppressed between the clamps, or with a pair offorceps or hooks, the edges may be drawn between them, and the screws turned so as to close upon the fistula. The handle of the right hand screw, or one-half of its entire length, is made movable so as to be detached, to give room for manipulation. The lips of the fistula are now denuded with the knife or scissors, pins thrust through them, ligatures twisted around their points and heads, the instrument unscrewed and removed, and the operation will have been completed.

I prefer the figure of 8 suture, and it may be tightend after the screw clamp has been removed. The points of the pins should be cut off, and they themselves may be prevented ulcerating the vagina by the interposition of some soft material. The ligature may be thread or wire, and a single loop will answer every purpose. Probably silk would be best.

The advantages proposed by this new operation are:

It may be performed in fifteen or twenty minutes; an important consideration when we recollect the position of the patient on her knees and elbows.

The fistula is fully exposed.

Hæmorrhage is prevented by the pressure of the instrument.

We avoid wounding the bladder.

Broad surfaces can brought in apposition.

The line of union is seen throughout the treatment.

The after treatment may not be as rigorous as that now recommended. The bowels may be moved after the second or third day, and then kept in a laxative condition; the patient may begin early to use the catheter herself at short intervals, and her diet may be a little more generous. The horizontal position should, however, be observed most of the time.

The apparatus is readily removed, the instrument itself, and then the sutures simply by withdrawing the pins. Who, let me ask, has not experienced difficulty in detaching the suture, in some cases so great as even to interrupt the healing of the fistula?

It is comparatively free of danger.

The method is far less painful. In my six cases but ten attempts, every patient has complained most distresingly of the Sims' and Bozeman's operation.

Though not adapted to all cases, this operation no doubt can be applied to the majority of them.

I leave to others willingly, who may feel disposed to try the plan here described, to determine its value, or it may be to improve upon it.

Mr. Schott of this city, makes the screw-clamp for five dollars.

